# High EASIX score is an independent predictor of non-relapse mortality in patients with CMML undergoing allogeneic stem cell transplant

**DOI:** 10.1038/s41409-022-01829-w

**Published:** 2022-09-21

**Authors:** Anmol Baranwal, Abhishek Mangaonkar, Mithun V. Shah, Mark R. Litzow, William J. Hogan, Mrinal M. Patnaik, Hassan B. Alkhateeb

**Affiliations:** Division of Hematology, Department of Medicine, Mayo Clinic, Rochester, MN USA

**Keywords:** Medical research, Health care

## To the Editor:

Chronic myelomonocytic leukemia (CMML) is a chronic, clonal disorder, of monocytes. A diagnosis of CMML requires that monocytes comprise at least 10% of the peripheral blood white blood cell differential with a sustained absolute monocyte count of ≥1 × 10^9^ cells/L, and the absence of other disease-defining genetic abnormalities, such as *BCR-ABL1*, *PDGFRA*, *PDGFRB*, *FGFR1*, or *PCM1-JAK2* fusions [1].

Allogeneic stem cell transplant (alloSCT) remains the only curative option for patients with CMML and studies evaluating alloSCT outcomes have shown treatment-related mortality ranging from 12 to 52% [2–8]. It was recently shown that the endothelial activation and stress index (EASIX) score could also predict mortality after alloSCT [9]; however, its value is limited to AML and MDS.

The goal of our study was to assess if pre-conditioning EASIX can predict non-relapse mortality (NRM) in patients with CMML undergoing alloSCT.

**Fig. 1:**
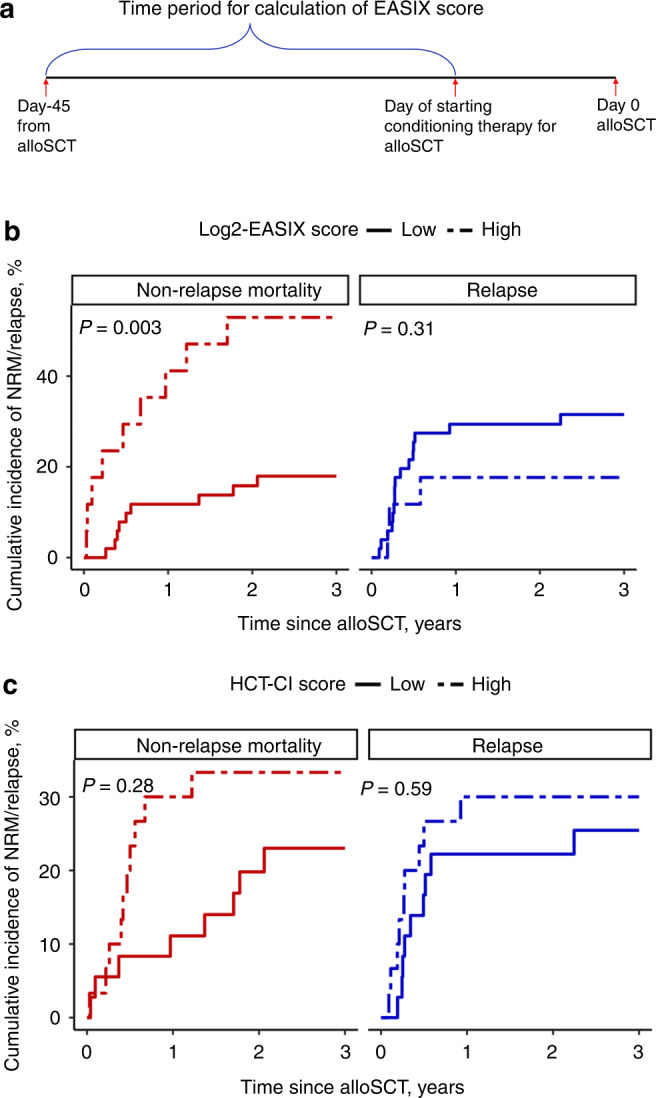
Calculation of EASIX score and competing risk analyses. **a** Time period for calculation of EASIX score. **b** Non-relapse mortality and relapse stratified by low and high log_2_-EASIX scores. **c** Non-relapse mortality and relapse stratified by low and high HCT-CI scores.

Patients with CMML who underwent an alloSCT at Mayo Clinic between November 1992 and October 2021 were included. EASIX score was calculated using the formula: lactate dehydrogenase [LDH (U/L)] × Creatinine (mg/dL) / platelet count (10^9^/L) and analyzed based on log_2_-transformed values. The LDH, creatinine, and platelet values available on the day of or prior to starting conditioning therapy, within 45 days of alloSCT, were used for calculation of the EASIX score (Fig. 1a). We divided the log_2_-EASIX scores into quartiles and evaluated the log_2_-EASIX quartile cut-offs for their impact on NRM. Patients with an HCT-CI score ≥3 were considered to have high HCT-CI scores. The cumulative incidence of NRM was determined using competing risk analyses, with relapse considered as a competing risk event. Further details of the method description are provided in the Supplementary materials.

A total of 68 patients (68% males) were evaluated for the purposes of the study. Median age at diagnosis was 60 years (range 18–73 years). Thirty-two (47.1%) patients had dysplastic subtype of CMML, while 36 (52.9%) patients had proliferative type of CMML.

Seventeen (25%) patients had progressed to CMML blast phase before alloSCT. A total of 57 (83.8%) patients had received some treatment for CMML before alloSCT (Supplementary Table 1). Eighteen (26.5%) patients were in complete remission at the time of alloSCT.

At transplant, the median age for the entire cohort was 61 years (range: 18–73 years). The median time of follow-up was 5.5 years (95% CI 5–12.3 years) and at last follow-up a total of 25 (36.8%) patients were alive. The median OS was 3.2 years (95% CI 1.37–13.6 years). The cumulative incidence of NRM for the entire cohort was 7.4%, 19.1%, 26.7% at 100 days, 1 year, and 3 years post alloSCT, respectively. The median log_2_-EASIX score was 0.89 (range −2.29 to 5.91). Upon analyzing log_2_-EASIX values by quartiles, the 75th percentile log_2_-EASIX score of 2.32 most optimally correlated with 3-year NRM and OS post alloSCT. Accordingly, 17 (25%) patients had a high log_2_-EASIX score and 51 (75%) had a low log_2_-EASIX score. While 26 (38.2%) patients had alloSCT before the year 2011, the proportion of patients with high EASIX scores was not significantly different between those undergoing alloSCT before or after the year 2011 (30.8% vs. 21.4%, *P* = 0.56) (Supplementary Table 2).

The cumulative incidence of NRM among patients with high EASIX score was significantly higher compared to those with low EASIX score at 100 days (23.5% vs. 2.0%, *P* = 0.003), at 1 year (41.2% vs. 11.8%, *P* = 0.007) and at 3 years (52.9% vs. 17.9%, *P* = 0.003) after alloSCT (Fig. 1b).

There was no significant difference in relapse rates between patients with high versus low EASIX score at 100 days (11.8% vs. 15.7%, *P* = 0.72) at 1 year (17.6% vs. 29.4%, *P* = 0.37) and at 3 years (17.6% vs. 31.5%, *P* = 0.31) after alloSCT (Fig. 1b). Graft-versus-host disease (3 patients, 17.6%) and infection (3 patients, 17.6%) were the most common causes of NRM among patients with high EASIX scores (Supplementary Table 3).

A total of 66 patients were evaluated for HCT-CI score, of whom 30 (45.5%) had a high HCT-CI score. The median HCT-CI score was 2 (range: 0–8). NRM after alloSCT among patients with high HCT-CI score was not statistically different compared to those with low HCT-CI scores at 100 days (10% vs. 5.6%, *P* = 0.51), at 1 year (30% vs. 11.1%, *P* = 0.06), and at 3 years (33.3% vs. 23%, *P* = 0.28) after alloSCT (Fig. 1c).

Patients with a high log_2_-EASIX score had a worse 3-year OS (29.4% vs. 57.4%, *P* = 0.017, Supplementary Fig. 1a), further reflecting the significant association of high EASIX score with NRM. There was a trend towards worse 3-year OS among patients with high compared to low HCT-CI scores (3-year OS rate 39% vs. 59.4%, *P* = 0.056, Supplementary Fig. 1b).

The univariate cox-proportional hazard model showed that an increasing log_2_-EASIX score, when analyzed as a continuous variable, adversely affected the 3-year post-alloSCT survival (HR 1.41, 95% CI 1.1–1.81, *P* = 0.007).

High (vs. low) EASIX score, progression to blast phase before alloSCT, non-matched-related donor, and major ABO mismatch were significantly (*P* < 0.10) associated with NRM in univariate analysis (Supplementary Table 4). The multivariate competing risk regression analysis confirmed that high EASIX score is an independent risk factor for NRM at 3 years post-transplant (HR 3.88, 95% CI 1.53–9.88, *P* = 0.004), in addition to major ABO mismatch, and non-matched-related donor (Supplementary Table 5).

Several prognostic scoring systems have been developed in CMML and have been shown to have comparable performance [10]. However, these prognostic models may have limited ability to predict NRM after alloSCT. For instance, in a study by Liu et al. a high CPSS score was associated with increased mortality in patients who had relapsed; however, it was not associated with NRM [11]. Likewise, the MD Anderson prognostic scoring was also not found to be associated with relapse-free survival post-transplant [12].

The 26.7% cumulative incidence of NRM in our cohort is parallel to what has been reported in other studies [2–8]. Our study shows that the EASIX score is a significant predictor of NRM after alloSCT. A high EASIX score pretransplant was able to predict NRM at 100 days, 1 year, and 3 years after alloSCT. The strength of EASIX score is its simplicity and ease of calculation. Therefore, EASIX calculated during pretransplant evaluation can be a simple, yet strong, tool to ascertain the risk of NRM.

Limitation of our study is that it is a retrospective analysis with a small sample size. However, given that CMML has a lower incidence, most of the single-institution studies are limited by a small sample size.

In summary, this is the first study assessing the predictive power of EASIX score for NRM in patients with CMML undergoing alloSCT. Our study suggests that a log_2_-EASIX score ≥2.32 is an independent predictor of increased risk of NRM after alloSCT, irrespective of HCT-CI score. Patients with high log_2_-EASIX score at pretransplant evaluation should be carefully evaluated due to the high risk of associated NRM after transplant. Due to the small sample size, larger retrospective studies are required to validate our findings.

## Supplementary information

Supplementary material

## Data Availability

The datasets generated during and/or analyzed during the current study are available from the corresponding author upon reasonable request.
