# Supplementary material for: High EASIX score is an independent predictor of non-relapse mortality in patients with CMML undergoing allogeneic stem cell transplant
Source: Bone Marrow Transplant. 2022 Sep 21;57(12):1842–4. doi: 10.1038/s41409-022-01829-w (PMC9715426; doi:10.1038/s41409-022-01829-w)
Supplement: Supplementary file 1 — Supplementary material [file 41409_2022_1829_MOESM1_ESM.docx]

**Supplement**

**Supplemental methods:**

*Patients:* Patients with CMML who underwent an alloSCT at Mayo Clinic between November 1992, to October 2021 were included in the study. This study was approved by the Mayo Clinic Institutional Review Board. We examined the Mayo Clinic database and the available electronic medical records to determine the following: patient, disease, and transplant characteristics: age at diagnosis, age at transplant, sex, CMML subtype, splenomegaly at diagnosis, progression to blast phase, disease status at time of alloSCT, HCT-CI score, conditioning regimen, conditioning intensity, graft source, donor-recipient sex match, and graft-versus-host disease (GVHD) prophylaxis. The following transplant associated outcomes were reviewed: time to disease relapse, non-relapse mortality, GVHD, and death. Patients with inadequate medical records were removed from the study.

Most of the studies evaluating alloSCT outcomes in patients with CMML have only included patients who were transplanted before the year 2011 (1–7). We understand that our patient cohort spans a period from 1992 to 2021. To avoid the time bias we also analyzed NRM outcomes between patients undergoing alloSCT from year 2011 onwards to those who underwent alloSCT before 2011.

Relapse was defined as detection of disease after alloSCT by morphological, cytogenetic, and genetic analysis as applicable. Intensity of conditioning regimens were defined per the CIBMTR definitions (8). Acute GVHD was graded according to Glucksberg criteria, and chronic GVHD was graded according to the 2014 National Institute of Health (NIH) consensus criteria (9,10).

EASIX score was calculated using the formula: lactate dehydrogenase [LDH (U/L)] × Creatinine (mg/dL) / platelet count (10^9/L) and analyzed based on log2 transformed values. The LDH, creatinine and platelet values available on the day of or prior to starting conditioning therapy, within 45 days of alloSCT, were used for calculation of the EASIX score. Similarly, HCT-CI score was calculated with values closest to the day of or prior to starting conditioning therapy.

*Statistical Methods:*

Data on patient-, disease- and transplant-characteristics, and post-transplant outcomes as mentioned above were collected retrospectively. Patient and transplant characteristics were summarized using descriptive statistics. The statistical comparison of categorical variables between patients with high *versus* low EASIX scores was performed using a chi-square test, and t-test was used for comparison of continuous variables.

We divided the log2-EASIX scores into quartiles and evaluated the log2-EASIX quartile cut-offs for their impact on non-relapse mortality. Patients with an HCT-CI score ≥ 3 were considered to have a high HCT-CI scores.

The cumulative incidence of NRM was determined using competing risk analyses, with relapse considered as competing risk event. Gray’s analysis was used to compare the differences between the cumulative incidence curves (11).

The cumulative incidence of NRM was determined at day 100, 1 year, and 3 years after alloSCT. Patients were censored at last known follow-up if they did not develop any of the competing risk events.

Overall survival from transplant was determined using Kaplan-Meier and log-rank method (12). Median follow-up time from transplant was calculated using the reverse Kaplan-Meier method (13). Cox-proportional hazard model was used to analyze the effect of EASIX score on post-alloSCT survival. Finally, we used Fine-Gray competing risk regression analyses to determine risk factors for NRM (14). To avoid time-bias, we also analyzed if patients who underwent alloSCT before year 2011 had a significantly different cumulative incidence of NRM compared to those had alloSCT since year 2011 onwards. Variables found significant in univariate analyses at level of significance ≤ 0.10 were included in the multivariate competing risk regression analysis to determine significant risk factors that lead to increased NRM in patients with CMML undergoing alloSCT.

All statistical analyses were performed using R (version 4.1.1; R Foundation for Statistical Computing, Vienna, Austria); level of significance was set at p < 0.05.

**Supplemental references:**

1. Mittal P, Saliba RM, Giralt SA, Shahjahan M, Cohen AI, Karandish S, et al. Allogeneic transplantation: a therapeutic option for myelofibrosis, chronic myelomonocytic leukemia and Philadelphia-negative/BCR-ABL-negative chronic myelogenous leukemia. Bone Marrow Transplantation. 2004 May 29;33(10):1005–9.

2. Elliott MA, Tefferi A, Hogan WJ, Letendre L, Gastineau DA, Ansell SM, et al. Allogeneic stem cell transplantation and donor lymphocyte infusions for chronic myelomonocytic leukemia. Bone Marrow Transplantation. 2006 Jun 10;37(11):1003–8.

3. Ocheni S, Kröger N, Zabelina T, Zander AR, Bacher U. Outcome of allo-SCT for chronic myelomonocytic leukemia. Bone Marrow Transplantation. 2009 Apr 10;43(8):659–61.

4. Krishnamurthy P, Lim ZY, Nagi W, Kenyon M, Mijovic A, Ireland R, et al. Allogeneic haematopoietic SCT for chronic myelomonocytic leukaemia: a single-centre experience. Bone Marrow Transplantation. 2010 Oct 25;45(10):1502–7.

5. Eissa H, Gooley TA, Sorror ML, Nguyen F, Scott BL, Doney K, et al. Allogeneic Hematopoietic Cell Transplantation for Chronic Myelomonocytic Leukemia: Relapse-Free Survival Is Determined by Karyotype and Comorbidities. Biology of Blood and Marrow Transplantation. 2011 Jun;17(6):908–15.

6. Park S, Labopin M, Yakoub-Agha I, Delaunay J, Dhedin N, Deconinck E, et al. Allogeneic stem cell transplantation for chronic myelomonocytic leukemia: a report from the Societe Francaise de Greffe de Moelle et de Therapie Cellulaire. European Journal of Haematology. 2013 May;90(5):355–64.

7. Itonaga H, Iwanaga M, Aoki K, Aoki J, Ishiyama K, Ishikawa T, et al. Impacts of graft-versus-host disease on outcomes after allogeneic hematopoietic stem cell transplantation for chronic myelomonocytic leukemia: A nationwide retrospective study. Leukemia Research. 2016 Feb;41:48–55.

8. Bacigalupo A, Ballen K, Rizzo D, Giralt S, Lazarus H, Ho V, et al. Defining the Intensity of Conditioning Regimens: Working Definitions. Biology of Blood and Marrow Transplantation. 2009 Dec;15(12):1628–33.

9. GLUCKSBERG H, STORB R, FEFER A, BUCKNER CD, NEIMAN PE, CLIFT RA, et al. CLINICAL MANIFESTATIONS OF GRAFT-VERSUS-HOST DISEASE IN HUMAN RECIPIENTS OF MARROW FROM HL-A-MATCHED SIBLING DONOR,S. Transplantation. 1974 Oct;18(4):295–304.

10. Jagasia MH, Greinix HT, Arora M, Williams KM, Wolff D, Cowen EW, et al. National Institutes of Health Consensus Development Project on Criteria for Clinical Trials in Chronic Graft-versus-Host Disease: I. The 2014 Diagnosis and Staging Working Group Report. Biology of Blood and Marrow Transplantation. 2015 Mar;21(3):389-401.e1.

11. Gray RJ. A Class of K-Sample Tests for Comparing the Cumulative Incidence of a Competing Risk. The Annals of Statistics [Internet]. 1988;16(3):1141–54. Available from: http://www.jstor.org/stable/2241622

12. Kaplan EL, Meier P. Nonparametric Estimation from Incomplete Observations. J Am Stat Assoc. 1958 Jun;53(282):457–81.

13. Shuster JJ. Median follow-up in clinical trials. Journal of Clinical Oncology. 1991 Jan;9(1):191–2.

14. Fine JP, Gray RJ. A Proportional Hazards Model for the Subdistribution of a Competing Risk. J Am Stat Assoc. 1999 Jun;94(446):496–509.

**Supplemental Table 1:** Patient and disease characteristics stratified by EASIX score.

|  | **Low log2-EASIX score (N=51)** | **High log2-EASIX score (N=17)** | ***P*** |
| --- | --- | --- | --- |
| **Age at alloSCT** |  |  |  |
| Mean (SD) | 57.4 (11.7) | 57.5 (11.9) | 0.98 |
| Median [Min, Max] | 60.5 [18.3, 73.5] | 62.7 [32.1, 70.4] |  |
| **Gender** |  |  |  |
| Female | 19 (37.3%) | 3 (17.6%) | 0.23 |
| Male | 32 (62.7%) | 14 (82.4%) |  |
| **CMML type (by blast%)** |  |  |  |
| CMML-0 | 20 (39.2%) | 8 (47.1%) | 0.63 |
| CMML-1 | 15 (29.4%) | 3 (17.6%) |  |
| CMML-2 | 16 (31.4%) | 6 (35.3%) |  |
| **CMML type (by WBC count)** |  |  |  |
| Dysplastic | 22 (43.1%) | 10 (58.8%) | 0.4 |
| Proliferative | 29 (56.9%) | 7 (41.2%) |  |
| **CMML blast phase before alloSCT** |  |  |  |
| No | 40 (78.4%) | 11 (64.7%) | 0.42 |
| Yes | 11 (21.6%) | 6 (35.3%) |  |
| **Splenomegaly at diagnosis** |  |  |  |
| No | 43 (84.3%) | 15 (88.2%) | 1 |
| Yes | 8 (15.7%) | 2 (11.8%) |  |
| **Prior therapy before alloSCT** |  |  |  |
| No | 6 (11.8%) | 4 (23.5%) | 0.37 |
| Yes | 45 (88.2%) | 12 (70.6%) |  |
| Missing | 0 (0%) | 1 (5.9%) |  |
| Abbreviations: alloSCT: Allogeneic stem cell transplantation, CMML: Chronic myelomonocytic leukemia, EASIX: endothelial activation and stress index, WBC: White cell count. | | | |

**Supplemental Table 2:** Transplant characteristics and outcomes stratified by EASIX score.

|  | **Low log2-EASIX score (N=51)** | **High log2-EASIX score (N=17)** | ***P*** |
| --- | --- | --- | --- |
| **HCT-CI** |  |  |  |
| 0 - 2 | 29 (56.9%) | 7 (41.2%) | 0.48 |
| $\geq3$ | 21 (41.2%) | 9 (52.9%) |  |
| Missing | 1 (2.0%) | 1 (5.9%) |  |
| **alloSCT from year 2011 onwards** |  |  |  |
| No | 18 (35.3%) | 8 (47.1%) | 0.56 |
| Yes | 33 (64.7%) | 9 (52.9%) |  |
| **Donor source** |  |  |  |
| Haploidentical | 3 (5.9%) | 1 (5.9%) | 0.55 |
| MMRD | 1 (2.0%) | 0 (0%) |  |
| MMUD | 2 (3.9%) | 0 (0%) |  |
| MRD | 19 (37.3%) | 6 (35.3%) |  |
| MUD | 26 (51.0%) | 9 (52.9%) |  |
| UCBT | 0 (0%) | 1 (5.9%) |  |
| **Graft source** |  |  |  |
| BMT | 7 (13.7%) | 3 (17.6%) | 0.19 |
| PBSCT | 0 (0%) | 1 (5.9%) |  |
| DUCBT | 44 (86.3%) | 13 (76.5%) |  |
| **Reduced-intensity conditioning** |  |  |  |
| No | 17 (33.3%) | 8 (47.1%) | 0.47 |
| Yes | 34 (66.7%) | 9 (52.9%) |  |
| **Busulfan-based conditioning** |  |  |  |
| No | 39 (76.5%) | 13 (76.5%) | 1 |
| Yes | 12 (23.5%) | 4 (23.5%) |  |
| **Major ABO mismatch** |  |  |  |
| No | 42 (82.4%) | 12 (70.6%) | 1 |
| Yes | 9 (17.6%) | 3 (17.6%) |  |
| Missing | 0 (0%) | 2 (11.8%) |  |
| **Both donor and recipient CMV negative** |  |  |  |
| Yes | 17 (33.3%) | 3 (17.6%) | 0.45 |
| No | 32 (62.7%) | 12 (70.6%) |  |
| Missing | 2 (3.9%) | 2 (11.8%) |  |
| **Acute GVHD grade 2-4** |  |  |  |
| No | 33 (64.7%) | 8 (47.1%) | 0.45 |
| Yes | 18 (35.3%) | 8 (47.1%) |  |
| Missing | 0 | 1 (5.8%) |  |
| **Acute GVHD grade 3-4** |  |  |  |
| No | 41 (80.4%) | 10 (58.8%) | 0.26 |
| Yes | 10 (19.6%) | 6 (35.3%) |  |
| Missing | 0 | 1 (5.9%) |  |
| **Chronic GVHD** |  |  |  |
| No | 18 (35.3%) | 9 (52.9%) | 0.23 |
| Yes | 33 (64.7%) | 7 (41.2%) |  |
| Missing | 0 (0%) | 1 (5.9%) |  |
| **Relapse** |  |  |  |
| No | 34 (66.7%) | 14 (82.4%) | 0.36 |
| Yes | 17 (33.3%) | 3 (17.6%) |  |
| **Non-relapse mortality** |  |  |  |
| No | 37 (72.5%) | 6 (35.3%) | 0.01 |
| Yes | 14 (27.5%) | 11 (64.7%) |  |
| Abbreviations: alloSCT: allogeneic stem cell transplant, EASIX: endothelial activation and stress index, GVHD: Graft-versus-host disease, HCT-CI: Hematopoietic Cell Transplantation-specific Comorbidity Index, MMRD: Mismatched related donor, MRD: matched related donor, MMUD: Mismatched unrelated donor, MUD: Matched unrelated donor, UCBT: umbilical cord blood transplant. | | | |

**Supplemental Table 3:** Causes of NRM by last follow-up stratified by EASIX score.

| **Causes of NRM** | **Low log2-EASIX score (N=51)** | **High log2-EASIX score (N=17)** |
| --- | --- | --- |
| Brain hemorrhage | 0 (0%) | 1 (5.88%) |
| Diffuse alveolar hemorrhage | 1 (1.96%) | 1 (5.88%) |
| Engraftment failure | 1 (1.96%) | 0 (0%) |
| Enterocolitis | 0 (0%) | 1 (5.88%) |
| GVHD | 3 (5.88%) | 3 (17.65%) |
| Infection | 1 (1.96%) | 3 (17.65%) |
| Multiorgan dysfunction | 2 (3.92%) | 0 (0%) |
| Second cancer | 2 (3.92%) | 0 (0%) |
| Unknown | 4 (7.84%) | 2 (11.76%) |
| Patients without NRM | 37 (72.55%) | 6 (35.29%) |
| Abbreviations: EASIX: endothelial activation and stress index, GVHD: graft-versus-host disease, NRM: non-relapse mortality. | | |

**Supplemental Table 4:** Univariate Fine-Gray completing risk regression analysis for non-relapse mortality at 3 years after alloSCT.

| **Characteristic** | **Hazard Ratio** | **95% CI** | ***P*** |
| --- | --- | --- | --- |
| Male gender | 0.56 | 0.22 – 1.38 | 0.21 |
| Age at alloSCT > 60 years | 0.88 | 0.35 – 2.17 | 0.77 |
| alloSCT from year 2011 onwards | 0.57 | 0.23 – 1.42 | 0.23 |
| Proliferative subtype | 0.5 | 0.20 – 1.27 | 0.14 |
| CMML-2 (vs CMML-1 + CMML-0) | 0.80 | 0.29 – 2.23 | 0.67 |
| Not CMML-0 | 1.89 | 0.66 – 5.38 | 0.23 |
| High EASIX score (log2-EASIX ≥ 2.32) | 4 | 1.61 – 9.92 | **0.003** |
| High HCTCI score (HCT-CI score ≥ 3) | 1.68 | 0.67 – 4.22 | 0.27 |
| PBSCT graft | 3.52 | 0.44 – 28.3 | 0.24 |
| RIC | 0.67 | 0.27 – 1.68 | 0.39 |
| Progression to blast phase before alloSCT | 2.22 | 0.87 – 5.66 | **0.096** |
| CR at alloSCT | 0.85 | 0.27 – 2.66 | 0.78 |
| Prior treatment before alloSCT | 3.34 | 0.45 – 24.8 | 0.24 |
| Donor type (vs matched-related donor) | 3.25 | 0.92 – 11.5 | **0.07** |
| Busulfan-based conditioning | 0.91 | 0.31 – 2.72 | 0.87 |
| Either donor/recipient CMV positive | 1.01 | 0.36 – 2.85 | 0.98 |
| Major ABO mismatch | 2.69 | 1.05 – 6.86 | **0.04** |
| Any ABO mismatch | 1.66 | 0.66 – 4.2 | 0.29 |
| Abbreviations: alloSCT: allogeneic stem cell transplant, AML: Acute Myeloid Leukemia, CI: Confidence interval, CMML: Chronic Myelomonocytic Leukemia, CMV: Cytomegalovirus, CR: Complete Remission, EASIX: Endothelial Activation and Stress Index, HCTCI: Hematopoietic Cell Transplantation-specific Comorbidity Index, MMUD: Mismatched unrelated donor, PBSCT: Peripheral blood stem cell transplant, RIC: Reduced intensity conditioning, UCB: Umbilical cord blood. | | | |

**Supplemental Table 5:** Multivariate Fine-Gray competing risk regression analysis for risk factors for non-relapse mortality at 3 years after alloSCT.

| **Variable** | **Hazard Ratio** | **95% CI** | ***P*** |
| --- | --- | --- | --- |
| High EASIX score (log2-EASIX ≥ 2.32) | 3.88 | 1.53 – 9.88 | 0.004 |
| Major ABO mismatch | 3.38 | 1.43 – 7.99 | 0.006 |
| Donor type (vs. matched-related donor) | 6.39 | 1.72 – 23.80 | 0.006 |
| Progression to blast phase before alloSCT | 2.41 | 0.95 – 6.11 | 0.06 |
| Abbreviations: alloSCT: allogeneic stem cell transplant, CI: confidence interval, EASIX: Endothelial Activation and Stress Index. | | | |

**Supplemental Figure 1.** Overall survival at 3 years. (A) Stratified by log2-EASIX score, (B) Stratified by HCT-CI score.

**
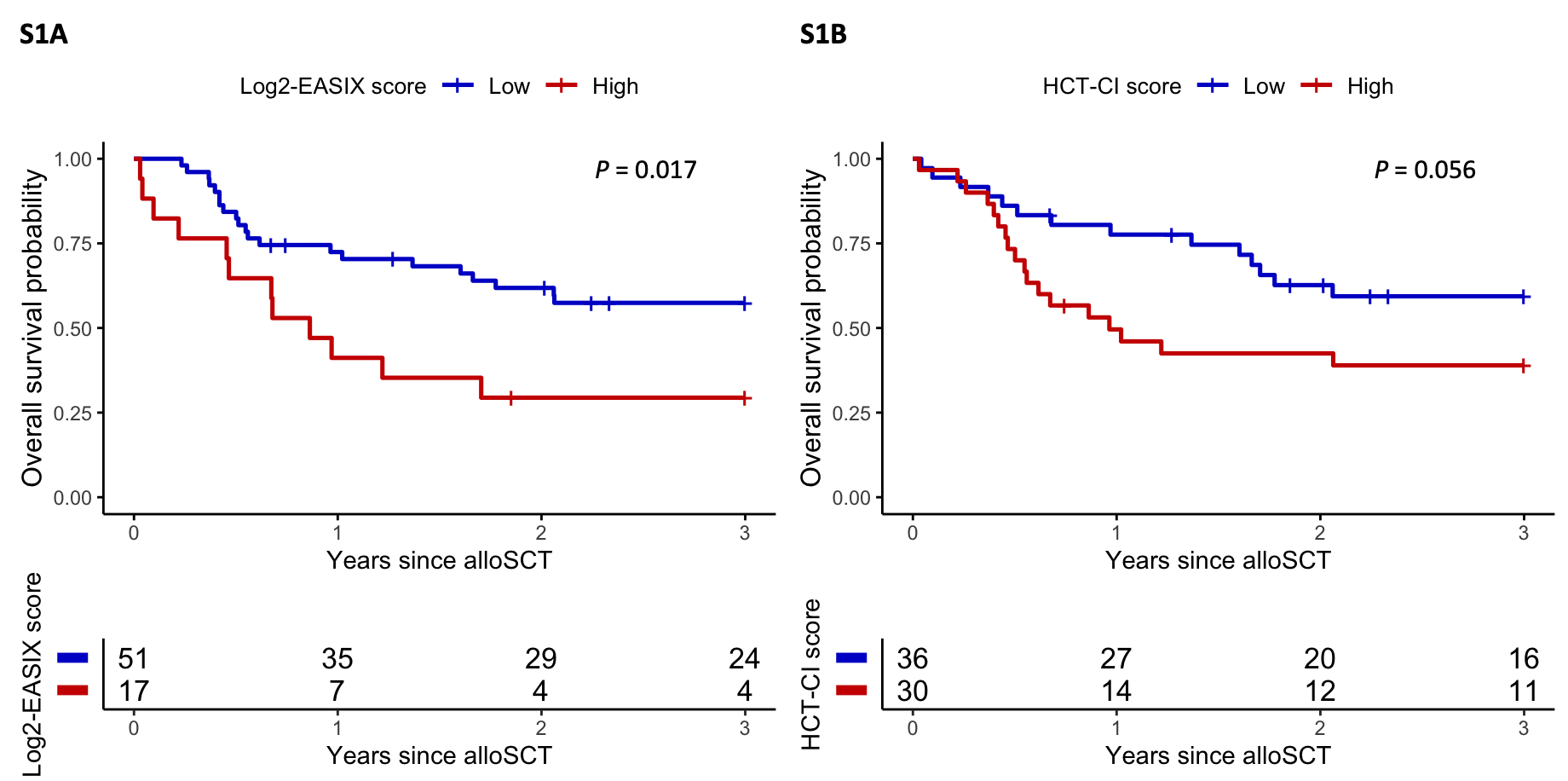
**
